# *Rhodosporidium* sp. DR37: a novel strain for production of squalene in optimized cultivation conditions

**DOI:** 10.1186/s13068-021-01947-5

**Published:** 2021-04-15

**Authors:** Shahryar Shakeri, Farshad Khoshbasirat, Mahmood Maleki

**Affiliations:** Department of Biotechnology, Institute of Science and High Technology and Environmental Sciences, Graduate University of Advanced Technology, Kerman, Iran

**Keywords:** *Rhodosporidium* sp. DR37, Lipid-based biofuels, Squalene production, Optimized medium

## Abstract

**Background:**

*Rhodosporidium* strain*,* a well-known oleaginous yeast, has been widely used as a platform for lipid and carotenoid production. However, the production of squalene for application in lipid-based biofuels is not reported in this strain. Here, a new strain of *Rhodosporidium* sp. was isolated and identified, and its potential was investigated for production of squalene under various cultivation conditions.

**Results:**

In the present study, *Rhodosporidium* sp. DR37 was isolated from mangrove ecosystem and its potential for squalene production was assessed. When *Rhodosporidium* sp. DR37 was cultivated on modified YEPD medium (20 g/L glucose, 5 g/L peptone, 5 g/L YE, seawater (50% v/v), pH 7, 30 °C), 64 mg/L of squalene was produced. Also, squalene content was obtained as 13.9% of total lipid. Significantly, use of optimized medium (20 g/L sucrose, 5 g/L peptone, seawater (20% v/v), pH 7, 25 °C) allowed highest squalene accumulation (619 mg/L) and content (21.6% of total lipid) in *Rhodosporidium* sp. DR37. Moreover, kinetic parameters including maximum specific cell growth rate (*μ*_*max*_, h^−1^), specific lipid accumulation rate (*q*_*p*_, h^−1^), specific squalene accumulation rate (*q*_*sq*_, h^−1^) and specific sucrose consumption rate (*q*_*s*_, h^−1^) were determined in optimized medium as 0.092, 0.226, 0.036 and 0.010, respectively.

**Conclusions:**

This study is the first report to employ marine oleaginous *Rhodosporidium* sp. DR37 for accumulation of squalene in optimized medium. These findings provide the potential of *Rhodosporidium* sp. DR37 for production of squalene as well as lipid and carotenoids for biofuel applications in large scale.

**Graphic abstract:**

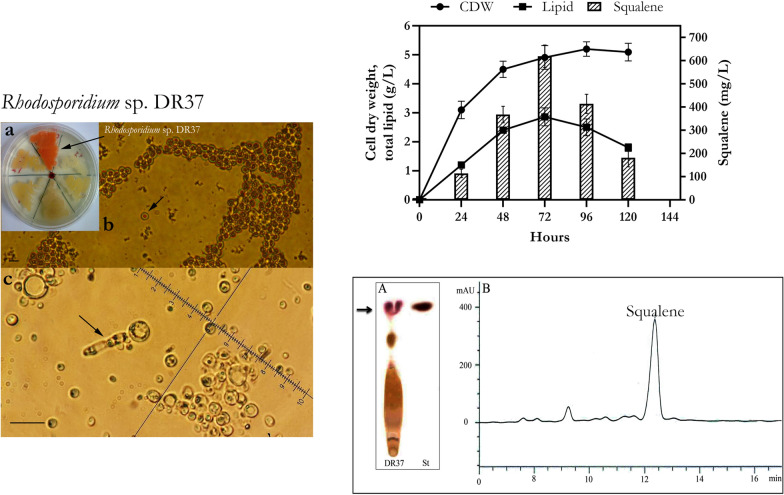

## Background

Squalene (2,6,10,15,19,23-hexamethyltetracosa-2,6,10,14,18,22-hexaene,), a six double-bonded triterpenic hydrocarbon (C_30_H_50_), is widely produced in microorganisms and the other higher organisms such as plants and animals via mevalonate (MVA) or 2-C-methyl-D-erythritol 4-phosphate (MEP) biosynthetic pathways [1]. Squalene is the main precursor of sterols biosynthesis in plants and animals [2]. It has been shown that squalene has antioxidant and anticancer activities with broad applications in food and cosmetics industries [3–5]. Besides, squalene has been used as vaccine adjuvant in vaccine formulations [6]. Recently, squalene has also been successfully converted to the biofuel and used as a diesel fuel by Mazda Motor Corporation (Fuchu, Japan) in 2011 [7]. Lipid-based biofuels have attracted more attention than petroleum-based fuels, after growing energy demand and global warming concerns [8]. Oleaginous microorganisms are promising sources for production of lipid-based biofuels [9]. For instance, oleaginous yeast strains can accumulate intracellular lipid up to 80% of their dry cell weight, which can be utilized for production of lipid-based biofuels [10]. Since, the demand for squalene has increased during the last decade, microbial production of squalene has been investigated as a promising alternative source for traditional extraction methods from shark liver or plant oils [11]. Microbial strains are capable of producing pollutant-free, low-cost, high-quality and sustainable squalene source as a major interest to lipid-based biofuels industries [12]. For these reasons, screening experiments and metabolic or bioprocess engineering have been used for isolation of naturally squalene producing microorganisms or improvement of squalene production in engineered microbes [11, 13, 14]. Squalene-producing microorganisms have been isolated and identified as yeast, fungi, bacteria, microalga and protists [15]. Recently, a protist (*Aurantiochytrium*) and a yeast (*Pseudozyma*) strain have been reported for their potential to produce high amount of squalene [16, 17]. *Aurantiochytrium* is known for its potential for production of high amount of polyunsaturated docosahexaenoic acid (DHA) in large scale [18]. Also, co-production of squalene and DHA has been reported by *Schizochytrium limacinum* SR21 from cheap feedstock of organosolv-pretreated spruce hydrolysates [19]. *Pseudozyma* is a biosurfactant, biodiesel and enzyme-producing yeast [20–22]. In recent years, high squalene producing yeast-like strains of *Pseudozyma* were isolated and identified, which are potential candidates for commercial production of squalene [16, 23].

This study has focused on oleaginous *Rhodosporidium* species which is basidiomycete, heterotrophic and fast growing yeast. *Rhodosporidium* accumulate high amount of intracellular lipid and carotenoids [24]. Till now, intensive researches have been done on production of lipid and carotenoids in this strain [25–28]. A wide range of carbon and nitrogen substrates utilization, fast growing and high content of lipid (30–70% of dry cell weight) and carotenoids such as β-carotene make *Rhodosporidium* useful for large-scale production of valuable metabolites [29]. Recently, *R. toruloides* DEBB 5533 has been used for pilot-scale oil production using sugarcane juice. Oil was produced with high productivity using fed-batch cultivation in bioreactor with working volume of 1000 L and then successfully converted to biodiesel with high performance in engine tests [30]. Also, genetic engineering of *Rhodosporidium* species for production of novel chemicals [31], in addition to co-production of carotenoids and lipid from cheap feedstocks [32], indicates that this strain is a promising candidate for production of high-value bioproducts. However, this biotechnologically important yeast strain is not being considered as a possible candidate for squalene production. Broad range substrate utilization and significant tolerance to biomass-derived inhibitors [33] make *Rhodosporidium* a promising candidate for economically feasible co-production of squalene with lipids, carotenoids and enzymes. Since, in carotenoid-producing yeasts, MVA pathway for production of carotenoids and squalene is the same, this possibility may exist to find some *Rhodosporidium* species with potential for production of squalene as much as carotenoids or lipids [24]. To the best of our knowledge, this study is the first effort using *Rhodosporidium* species for production of squalene. In this study, a newly isolated *Rhodosporidium* species was assessed for its potential for production of squalene. Furthermore, one factor at a time method was used for investigation of the individual effects of various factors (carbon and nitrogen sources, seawater concentration, pH and temperature) on cell growth, lipid and squalene production.

## Methods

### Materials and samples collection

All carbon and nitrogen sources were obtained from Merck (Darmstadt, Germany). Standard squalene was obtained from Sigma (St. Louis, Mo., U.S.A.). All other chemicals and solvents were provided in analytical grade. Seawater samples, fallen leaves of mangrove trees (*Avicennia marina* and *Rhizophora mucronata*) and sediments were collected from coastal waters and mangrove forest of Persian Gulf and Oman Sea in south of Iran (26°50′36.0"N 55°41′13.2"E and 26°19′30.9"N 57°07′28.0"E). Samples were kept in sterile bags at 4 °C and sent to the laboratory before use. Also, natural seawater sample with chemical composition including 7810 mg/L Na^+^, 22,961 mg/L Cl^−^, 410 mg/L Ca^2+^, 1386 mg/L Mg^2+^, 259 mg/L K^+^ and 2479 mg/L SO_4_^2−^ was used for preparation of culture media.

### Yeast strains isolation

Samples were plated on medium containing 10 g/L glucose, 1 g/L peptone, 0.1 g/L yeast extract (YE), natural seawater (50% v/v), 15 g/L agar and supplemented with 300 mg/L streptomycin and penicillin G. The plates were incubated at 30 °C for 3–6 days with regular observation for yeast growth. The obtained single yeast colonies were picked-up and sub-cultured on fresh yeast extract–peptone–dextrose medium (YEPD) (20 g/L glucose, 20 g/L peptone, 10 g/L YE, 15 g/L agar, seawater (50% v/v) and pH 6.5) and incubated at 30 °C for 48 h to obtain pure cultures [34].

### Light microscopy and morphological characteristics

Yeast strains were grown in YEPD broth medium and incubated at 30 °C for 48 h. Then, morphological characteristics of the isolated yeast strains were determined under light microscope (Zeiss, Germany).

### Molecular and phylogenetic analysis

An overnight culture of yeast strain was prepared and used for genomic extraction. Universal primers ITS1 (5´-TCCGTAGGTGAACCTGCG-3´) and ITS4 (5´-TCCTCCGCTTATTGATATGC-3´) were used for amplification of the internal transcribed spacer (ITS). Polymerase chain reaction (PCR) was performed by gradient thermal cycler (Eppendorf, Westbury, NY, USA). The program was set as denaturation for 4 min at 94 °C, 30 cycles of 30 s at 94 °C, 1 min at 55 °C and 90 s at 72 °C. Final extension was 7 min at 72 °C [35]. PCR amplicons were purified and sequenced by Bioneer. Resulting sequences were edited by BioEdit program (BioEdit 7.2) and were searched using Basic Local Alignment Search Tool (BLAST) (http://blast.ncbi.nlm.nih.gov). ClustalX program was used for sequence alignment and then generation of phylogenetic tree was done using neighbor-joining method and MEGA4 software [36]. The tree reliability was evaluated by bootstrap analysis of 1000 replicates.

### Strain selection and cultivation

Strain DR37 was isolated based on its characteristic for squalene production. This strain was cultivated in modified YEPD broth medium (20 g/L glucose, 5 g/L peptone, 5 g/L YE, seawater (50% v/v) and pH 7) and incubated at 30 °C and 150 rpm.

### Optimization of culture conditions

For investigation the individual effect of various chemical and physical parameters on cell growth, lipid and squalene production by *Rhodosporidium* sp. DR37, six sets of one factor at a time experiments were designed and previous optimum results were used for subsequent experiments. The first step to start the one factor at a time experiments was to find the optimum sole carbon source. Five carbon sources (20 g/L) (glucose, sucrose, glycerol, starch and olive oil) were tested for their effect on cell growth, lipid and squalene production. The next step was investigation of the effect of five nitrogen sources (5 g/L) (YE, malt extract (ME), peptone, ammonium chloride and sodium nitrate) with sucrose as the best sole carbon source. Seawater concentration (50% v/v) was constant in all above experiments. During carbon source experiments, YE and peptone (5 g/L each) were used as nitrogen source. Also, 20 g/L sucrose was used as the carbon source during nitrogen source experiments. Afterward, the effect of different concentrations of seawater (0, 20, 50, 70 and 100% v/v) was assessed along with 20 g/L sucrose and 5 g/L peptone as selected carbon and nitrogen sources, respectively. Also, different concentrations of selected carbon source (sucrose, g/L) (10, 20, 40, 60, 80 and 100) were investigated for their effect on cell growth, lipid and squalene production. Accordingly, the effects of various growth parameters, including temperature (15, 20, 25, 30 and 37 °C) and initial medium pH (5.0, 7.0 and 9.0) were investigated in medium containing 20 g/L sucrose, 5 g/L peptone and seawater (20% v/v). Initial pH of the medium was adjusted to 5.0, 7.0 and 9.0 by using hydrochloric acid and sodium hydroxide solutions. *Rhodosporidium* sp. DR37 was cultured in 250-mL Erlenmeyer flasks containing 50 mL broth fermentation medium. The experiments were carried out in triplicate and incubated for 3 days at 150 rpm [16, 26].

### Determination of cell growth and biomass

The growth of yeast cells were determined by measuring optical density (OD) of harvested cells at phosphate buffered saline (PBS) and 600 nm. Cell biomass, expressed as cell dry weight (CDW) was harvested by centrifugation (1300 g for 5 min) and pellets were dried at 105 °C for 18 h to constant weight and the weight was determined gravimetrically [18].

### Lipid extraction and TLC analysis

A certain amount of freeze-dried cells was suspended in 2 mL distilled water in screw cap test tubes. The cells were disrupted by ultrasonication (Hielscher UP200S, Germany; amplitude 60% and 0.5 cycle for 5 min) and then 2.5 mL chloroform and 5 mL methanol were added. Cell suspension was sonicated again and homogenized with T10 basic IKA homogenizer. 2.5 mL chloroform and 2.5 mL distilled water were added and vortexed for 30 s. Resulting suspension was centrifuged at 1300 *g* for 15 min to separate two phases. The organic bottom layer was transferred to a pre-weighed tube and the solvent was evaporated. The amount of lipid was determined gravimetrically [37]. Thin layer chromatography (TLC) silica gel plates coated with fluorescent indicator F_254_ were used for analysis of extracted lipids. Hydrocarbons such as squalene were separated with developing solvent of hexane/chloroform (9:1). Afterward, the TLC plates were exposed to H_2_SO_4_ (20%) and then visualized by heating at 70 °C for 60 min [38].

### Fatty acid methyl esters analysis

Total lipid was converted into fatty acid methyl esters (FAMEs) by methanolic sulfuric acid (4% v/v) at 80 °C for 90 min in sealed vials. Then 1 mL of H_2_O was added and FAMEs were extracted by hexane extraction (3 × 2 mL). FAMEs were dried over anhydrous Na_2_SO_4_ and solvent was removed by evaporation. The samples were stored at 4 °C prior analysis. Gas chromatography (GC) analysis was performed using Agilent 6890 equipped with a flame-ionization detector (FID) and DB-23 (30 m × 0.32 mm, 0.25 µm; Agilent Technologies, USA) capillary column. 0.5 µL of FAMEs sample was injected under splitless injection mode. Nitrogen was used as carrier gas and temperature of injector and detector was set at 300 °C. Column temperature program were 50 °C; 2 min, 10 °C/min to 180 °C; 5 min, 5 °C/min to 240 °C; 7 min. C19:0-FAME (Sigma, USA) was used as an internal standard [39].

### Quantitative determination of squalene by HPLC analysis

Squalene was identified and quantified by high-performance liquid chromatography (HPLC) (Agilent, 1100 Series, USA) equipped with a Zorbax, sb-C18 (4.6 × 250 mm, 5 micron) column. Lipids were saponified using 0.5 M potassium hydroxide containing ethanol (0.5 M KOH/EtOH) at 90 °C for 1 h. Then non-saponifiable lipids were extracted with hexane. Afterward, solvent was evaporated and squalene redissolved with 1 mL of acetonitrile/tetrahydrofuran (THF) (9:1, v/v) [13]. Acetonitrile/THF (80:20, v/v) was considered as mobile phase at a flow rate of 1 mL/min and ran under isocratic conditions. The sample injection volume was 10 μL and the column temperature was set at 30 °C and identification and quantification were done at 210 nm. Squalene (St. Louis, Mo., U.S.A.) was used as external standard for squalene quantification. A standard calibration curve was established by plotting peak area against concentration, by using different concentrations of squalene [40].

### Fourier-transform infrared spectroscopy (FT-IR) analysis

IR spectrum of purified squalene was determined between 4000 and 400 cm^−1^ using a Bruker ALPHA FT-IR spectrometer. Three spectral replicates were determined for purified squalene sample.

### Kinetic parameters calculation

The maximum specific cell growth rate (*μ*_*max*_, h^−1^), specific lipid accumulation rate (*q*_*p*_, h^−1^), specific squalene accumulation rate (*q*_*sq*_, h^−1^) and specific sucrose consumption rate (*q*_*s*_, h^−1^) were determined according to Ji et al. [41]. The growth yield (*Y*_*x/s*_, %), lipid yield (*Y*_*p/s*_, %), and squalene yield (*Y*_*sq/s*,_ %) were calculated according to Ren et al. [42], where x, p, sq and s are the amount (g/L) of biomass, lipid, squalene and residual sucrose, respectively.

### Statistical analysis

Analysis of variance (ANOVA) was used for data analysis with GraphPad Prism 8.0.1 (*P* < 0.05 was used for significant difference among means). The results are presented as the mean ± standard deviation of three replicates.

## Results and discussion

### Isolation and identification of ***Rhodosporidium*** sp. DR37

**Fig. 1:**
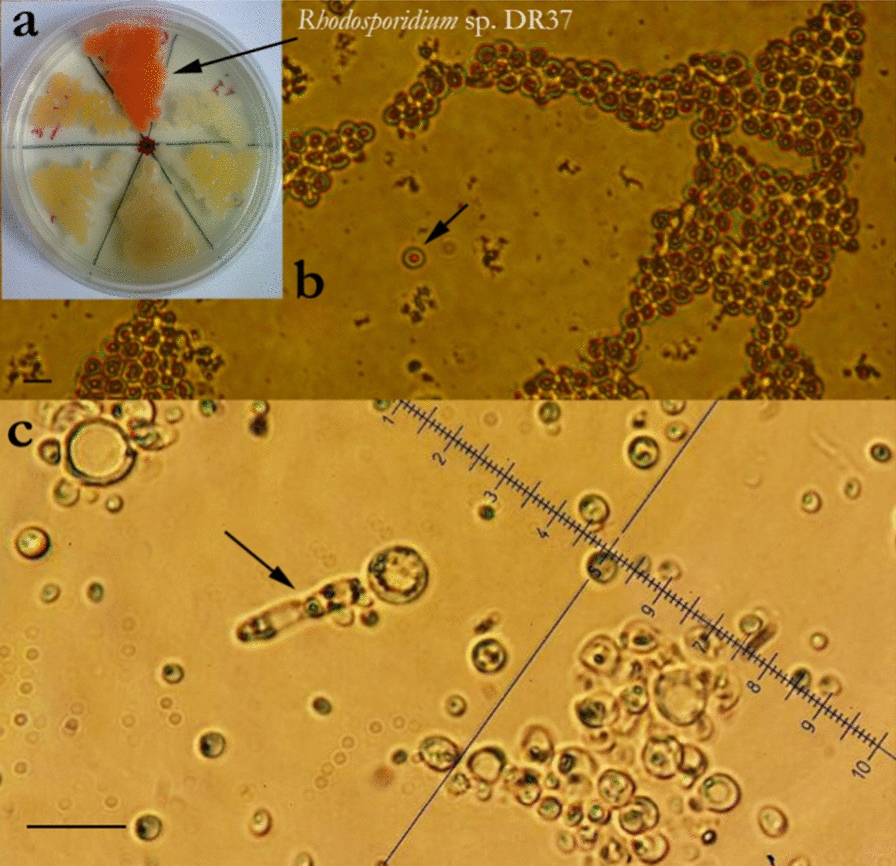
Morphological characteristics of strain DR37 (scale bar 10 μm). **a** Red colonies of strain DR37. **b** Yeast phase and a single cell containing red pigment (arrow), carotenoids are well shown as intracellular red pigments in yeast phase. **c** Elongated basidium (arrow) formed from germination of teliospore

In this study, a novel strain of *Rhodosporidium* was isolated and identified which is able to produce and accumulate large amount of intracellular lipid granules rich in squalene. Strain DR37 was isolated from water samples obtained from mangrove ecosystem of Qeshm, Iran. Morphology of DR37 colonies was observed as smooth, red, convex and round on the YEPD agar medium (Fig. 1a). Light microscopy showed single cells of DR37 containing red pigment and possess size ranging from 2 to 8 μm (Fig. 1b, arrow). Two phases of yeast-like and dikaryotic filamentous were observed after 20 days of incubation at 30 °C (Fig. 1c, arrow). As shown, arrow indicated an elongated basidium formed from germination of teliospore. Haploid basidiospores will emerge, germinate and form yeast phase of *Rhodosporidium* species [43]. The teleomorphic reproduction involves a transition between yeast phase and dikaryotic filamentous phase. These two phases are common features of *Rhodosporidium* species in sexual reproduction cycle [44].

### Phylogenetic analysis of strain DR37

**Fig. 2:**
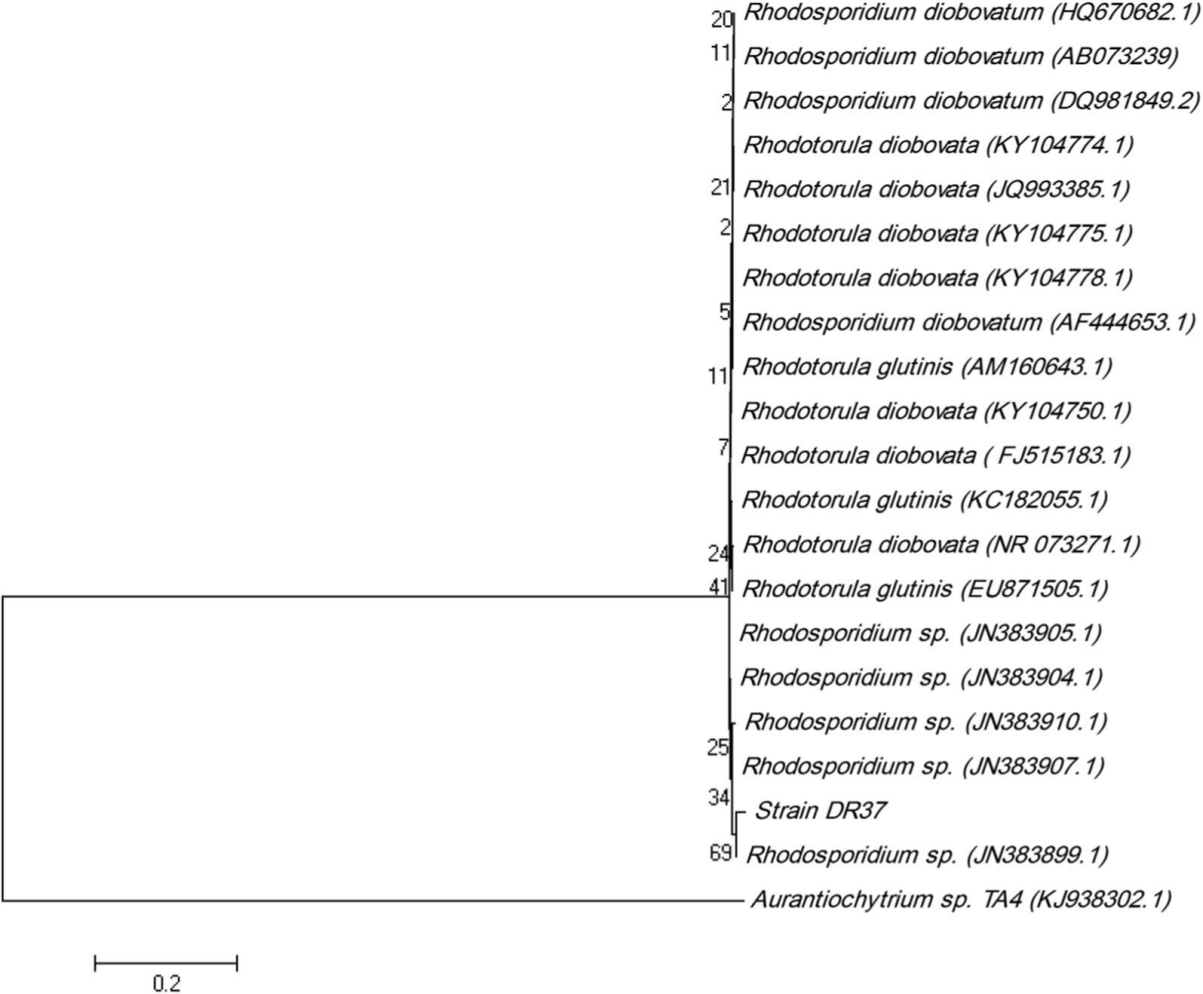
The phylogenetic tree of *Rhodosporidium* sp. DR37. Nucleotide sequences of ITS1, 5.8S rRNA, ITS2 were sequenced and tree was generated by neighbor-joining methods with bootstrap values (> 50%) from 1000 replicates. *Aurantiochytrium* sp. TA4 was considered as out-group

Phylogenetic tree of strain DR37 was generated by Mega × based on ITS1, 5.8S rRNA and ITS2 regions. Phylogenetic analysis confirmed *Rhodotorula* and *Rhodosporidium* species were the closest relatives of strain DR37 and indicated strain DR37 belongs to the genus of *Rhodotorula* (Fig. 2). These results were in agreement with data obtained after major revision on subphylum *Pucciniomycotina* published by Wang et al. [45]. It is shown that *Rhodosporidium* species is categorized under the revised *Rhodotorula* genus [46]. Phylogenetic analysis showed nucleotide sequences of ITS1, 5.8S rRNA and ITS2 from DR37 had 98% similarity to the species of *Rhodosporidium* JN383899. However, the main difference between *Rhodosporidium* and *Rhodotorula* species is the presence or absence of sexual reproduction, respectively. As a result, formation of basidium was observed by strain DR37 which is a part of sexual reproduction of *Rhodosporidium* species. Subsequently, based on morphological and phylogenetic characteristics, strain DR37 was named *Rhodosporidium* sp. DR37 with GenBank accession number MG022778.

### Determination of fatty acid profile of *Rhodosporidium* sp. DR37

*Rhodosporidium* sp. DR37 was cultivated in modified YEPD broth medium for 72 h. Lipid was extracted and subsequently esterified and analyzed by GC. *Rhodosporidium* sp. DR37 produced 5.2 g/L CDW and 0.46 g/L lipid. GC results showed that profile of fatty acids produced by *Rhodosporidium* sp. DR37 was similar to the other strain of *Rhodosporidium* TJUWZ4 [26]. The major fatty acids (% of TFA) were identified as C14:0 (myristic acid, 1.84%), C16:0 (palmitic acid, 26.22%), C16:1 (palmitoleic acid, 1.87%), C18:0 (stearic acid, 6.49%), C18:1 (oleic acid, 47.16%) and C18:2 (linoleic acid, 12.91%) (Table 1). Total content of saturated and unsaturated fatty acids was 34.55% and 61.94% of TFA, respectively. Oleic acid, palmitic acid and linoleic acid were predominant fatty acids with 86.29% of TFA. Higher content of these fatty acids among other fatty acids makes *Rhodosporidium* oil as a good feedstock for production of biodiesel with desirable properties [47]. The obtained profile of fatty acids from *Rhodosporidium* showed a similar composition to fatty acids obtained from plant oils and can be considered as a microbial alternative for production of biodiesel [48, 49]. Similar to *Rhodosporidium* sp. DR37, oleic acid was produced with high content among other fatty acids in total lipid (53.34%) by *Rhodosporidium toruloides* 21167 in a medium containing cassava starch hydrolysate at shake flask. It is notable that long-chain fatty acids containing of 16 and 18 carbon atoms are very desirable for biodiesel production [50].

**Table 1:** Fatty acid profile of *Rhodosporidium* sp. DR37

Fatty acids	(% of TFA)
C14:0	1.84
C16:0	26.22
C16:1	1.87
C18:0	6.49
C18:1	47.16
C18:2	12.91
Others	3.51
Total	100

### TLC analysis of total lipids

**Fig. 3:**
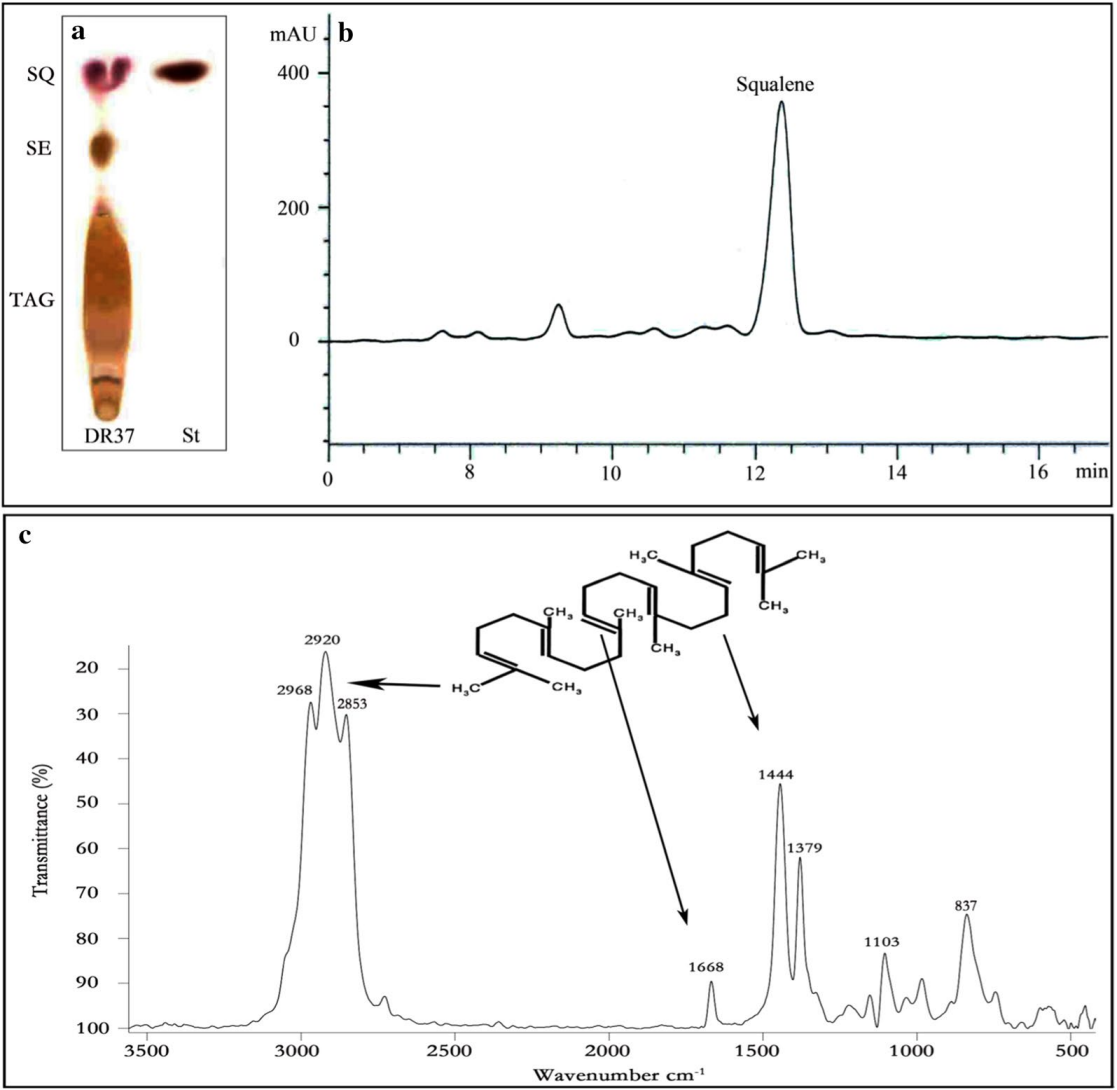
** a** TLC analysis of total lipid extracted from biomass of *Rhodosporidium* sp. DR37. Standard squalene was used as control for identification of squalene in the samples. **b** HPLC chromatogram of non-saponifiable lipid extract (squalene) from *Rhodosporidium* sp. DR37 oil. **c** The infrared (IR) spectrum of purified squalene. SQ squalene, TAG triacylglycerol, SE steryl esters

Figure 3a shows TLC of total lipid extracted from biomass of *Rhodosporidium* sp. DR37. Total lipids were extracted from *Rhodosporidium* and subjected to TLC and then, squalene and triacylglycerol (TAG) were separated by developing solvent. After visualization step, squalene spot was identified based on standard squalene. The RF value of visualized squalene spot from *Rhodosporidium* sp. DR37 was same as the standard squalene. TLC screening of squalene production among microbial strains is a simple and fast method in comparison to chromatography methods such as GC and HPLC [13]. As shown in Fig. 3a, TAG and steryl esters (SE) are recognized together with squalene which is in accordance with results of Milla et al. [51]. In another study, Patel et al. analyzed total lipid extract from *Aurantiochytrium* sp. T66 (ATCC PRA-276) by TLC. The two spots of SE and TAG were detected and recognized together with the squalene spot [52].

### Quantitative determination of squalene by HPLC

Further purification of squalene was performed by saponification of total lipid and then squalene extraction was carried out by *n*-hexane from non-saponifiable fraction. Detection and quantitative evaluation of purified squalene was performed by HPLC. Chromatographic peak of squalene was identified based on the retention time of standard squalene. As shown in Fig. 3b, the elution time for extracted squalene was 12.37 min. Then, squalene concentration was determined based on peak area and using a calibration curve. Squalene was produced at concentration of 64 mg/L and content of 13.9% of total lipid after 72 h of *Rhodosporidium* sp. DR37 cultivation in modified YEPD medium. *Rhodosporidium* strains are well known for their potential for production of carotenoids and lipids in their CDW. However, squalene content has been reported to be very low in *Rhodosporidium* strains in comparison to genetically modified yeasts or naturally squalene producing microorganisms such as *Schizochytrium*, *Aurantiochytrium* and *Pseudozyma*. A genetically modified *Saccharomyces cerevisiae* (strain YUG37-*ERG1*) was reported to accumulate 18.0 mg/L squalene [53] which is much lower than naturally produced squalene (64 mg/L) by *Rhodosporidium* sp. DR37. Kaya et al. reported an *Aurantiochytrium* strain which accumulates high amount of squalene with concentration of 1.29 g/L after 4 days of cultivation [38]. Also, another extended screening program was performed for isolation of squalene producing thraustochytrid strains in Okinawa mangrove forest. 14 novel squalene producing thraustochytrid strains were isolated and identified. The amount of produced squalene was determined between 7.54 and 13.9 g/g CDW [54].

### FT-IR analysis of squalene

The IR spectrum of squalene is shown in Fig. 3c. Three intense bands corresponding to C–H stretching were observed at 2968, 2920 and 2853 cm^−1^. Furthermore, four intense skeletal vibration modes were observed at 1444, 1379, 1103 and 837 cm^−1^. More importantly, one low-intensity band corresponding to C=C stretching was observed at 1668 cm^−1^. These IR results are completely in agreement with Hall et al. [55]. They found an analytically important band at 1666 cm^−1^ and 1670 cm^−1^ in FT-IR and Raman analysis of squalene, respectively. These bands corresponding to symmetric stretching of six double bonds in squalene structure can be used to discriminate squalene from TAG or diacylglycerol (DAG) in FT-IR or Raman analysis.

### The effect of carbon sources on cell growth, lipid and squalene production

**Fig. 4:**
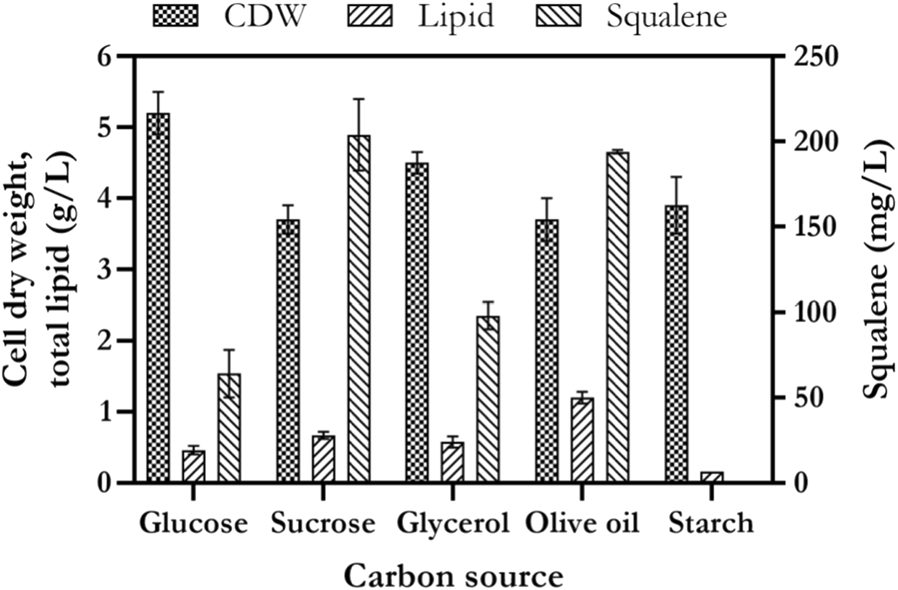
The effect of various carbon sources on cell growth, lipid and squalene production by *Rhodosporidium* sp. DR37 at 30 °C and 150 rpm. The samples were taken after 72 h of cultivation and cell growth, lipid and squalene production were assessed. The results are the mean ± standard deviation of three replicates

Optimum condition for squalene production was investigated by one factor at the time method. The cell growth, lipid and squalene production were assessed on various carbon and nitrogen sources and seawater concentrations under various temperatures and pH. Glucose, sucrose, glycerol, olive oil and starch were used to investigate their effect on cell growth, lipid and squalene production in shake flask. As shown in Fig. 4, *Rhodosporidium* sp. DR37 was able to grow on all carbon sources. The maximum CDW was obtained in medium with glucose as sole carbon source (5.2 g/L). *Rhodosporidium* sp. DR37 produced lipid in almost all carbon sources except in medium containing starch. This result was in agreement with previous data obtained for *Rhodosporidium* TJUWZ4 by Wang et al. [26]. Their results showed that *Rhodosporidium* TJUWZ4 produced very low lipid content (0.039 g/L) in medium containing starch as sole carbon source. No statistically significant difference was observed between CDW or squalene values obtained from medium containing sucrose or olive oil as sole carbon source (*p* < 0.05). Maximum lipid concentration of 1.2 g/L was achieved in medium containing olive oil as carbon source. However, sucrose was selected to be the best carbon source for squalene production. The squalene concentration was slightly higher in *Rhodosporidium* grown on sucrose (204 mg/L) in comparison to olive oil (194 mg/L) (*p* > 0.05). Therefore, sucrose was selected as the best carbon source for further optimization experiments. Sucrose is one of the main compositions of sugarcane molasses as a low-cost carbon source for production of high-added value bioproducts by microorganisms [56]. *Rhodosporidium* species is also capable of accumulating high amount of lipid from 5 carbon carbohydrates such as xylose, which is presented in plant biomass hydrolysates [57].

### The effect of nitrogen sources on cell growth, lipid and squalene production

**Fig. 5:**
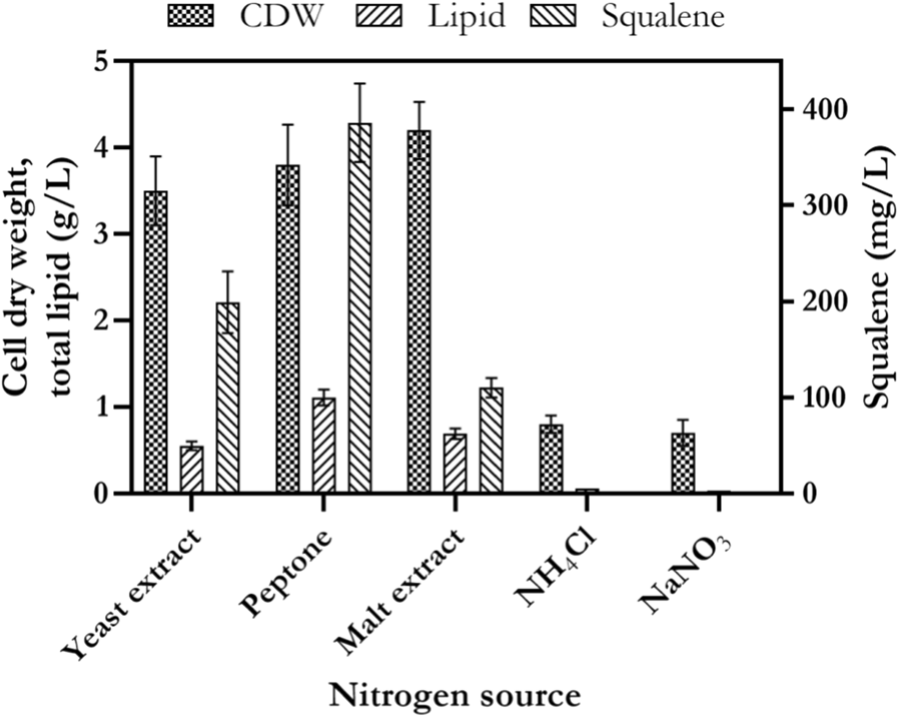
The effect of various nitrogen sources on cell growth, lipid and squalene production by *Rhodosporidium* sp. DR37 at 30 °C and 150 rpm

Five different nitrogen sources including organic (YE, peptone and ME) and inorganic (ammonium chloride, and sodium nitrate) were used to investigate their effect on biomass, lipid and squalene production. As shown in Fig. 5, results indicated YE, peptone and ME supported considerable cell growth in comparison to inorganic nitrogen sources. A significant difference was observed between *p* values obtained from organic and inorganic nitrogen sources (*p* < 0.05). Lipid concentration was 0.55, 1.11 and 0.69 g/L, when YE, peptone and ME were used as sole nitrogen source in culture medium, respectively. Among nitrogen sources, YE and peptone were found to have a statistically significant effect on squalene production (*p* < 0.05). The highest squalene concentration (386 mg/L) was obtained in culture medium containing peptone as sole nitrogen source. Squalene concentration was 199 and 110 mg/L, when YE and ME were used as sole nitrogen source in culture medium, respectively. Maximum amounts of lipid and squalene were obtained in culture medium with peptone as sole nitrogen source. These results showed that organic nitrogen sources such as peptone are effective in production of squalene by marine strain *Rhodosporidium* sp. DR37. Nitrogen sources are necessary for cell growth and maintenance of *Rhodosporidium* during exponential growth phase in the initial fermentation stage with balanced C/N ratio [29]. Peptones are derived by enzymatic digestion or acid hydrolysis of animal and plant tissues and usually used for enrichment of culture medium to promote cell growth and multiplication [58]. As shown in Fig. 5, organic nitrogen sources such as peptone and YE supported better cell growth, lipid and squalene production in comparison to inorganic nitrogen sources. The reason for this can be explained by composition of peptone or YE. Peptone and YE have a rich composition of amino acids, vitamins, organic and inorganic minerals to support cell growth and product formation [59]. On the other hand, inorganic nitrogen sources such as NH_4_Cl or NaNO_3_ may change some chemical parameters such as pH in broth medium and subsequently, cause negative effects on cell growth and product formation [60]. As mentioned above, maximum levels of CDW, lipid and squalene were produced by *Rhodosporidium* sp. DR37, when peptone was used as sole nitrogen source in culture medium. Besides, lipid accumulation in *Rhodosporidium* could be achieved by a proper carbon-to-phosphorus (C/P) ratio, regardless of high amount of nitrogen source in fermentation medium [61].

### The effect of salinity on cell growth, lipid and squalene production

**Fig. 6:**
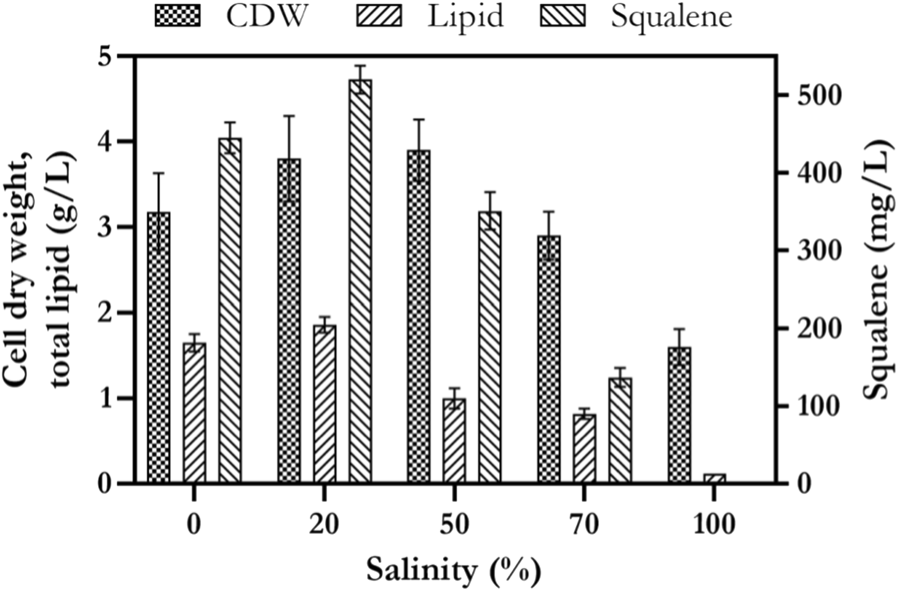
The effect of different seawater concentrations on cell growth, lipid and squalene production by *Rhodosporidium* sp. DR37 at 30 °C and 150 rpm

The effect of different concentrations of seawater (0, 20, 50, 70 and 100% v/v) was assessed on cell growth, lipid and squalene production by *Rhodosporidium* sp. DR37, in culture medium containing 20 g/L sucrose and 5 g/L peptone as selected carbon and nitrogen sources, respectively. As shown in Fig. 6, *Rhodosporidium* sp. DR37 was capable of growing and producing biomass between 1.6–3.9 g/L in medium containing 0–100% seawater concentration. Addition of 20% v/v seawater to the culture medium, resulted in production of lipid at its maximum level (1.86 g/L) in comparison to other media. Lowest amount of lipid production was observed in medium with 100% seawater strength. In fact increasing of seawater concentration showed a statistically significant negative effect (*p* < 0.05) on lipid production by *Rhodosporidium* sp. DR37. The maximum level of squalene (520 mg/L) was obtained in medium with 20% v/v seawater and then the amount of squalene was significantly decreased to its minimum level in medium with 70% v/v seawater (*p* < 0.05). Neither lipid nor squalene, produced in 100% seawater concentration. The results of Tchakouteu et al. [62] research work showed that *Rhodosporidium toruloides* DSM 4444 was able to tolerate salinity and grow well in medium containing 6% w/v NaCl. The presence of high level salt in culture medium was reported as a good factor for prevention of microbial contaminations in fermentation broth. Interestingly, their results showed that lipid accumulation was stimulated in medium with 4% w/v NaCl. In our study, *Rhodosporidium* sp. DR37 was able to grow in medium containing 100% v/v seawater. However, the maximum levels of lipid and squalene were obtained in medium containing 20% v/v seawater.

### The effect of different concentrations of sucrose on cell growth, lipid and squalene production

**Fig. 7:**
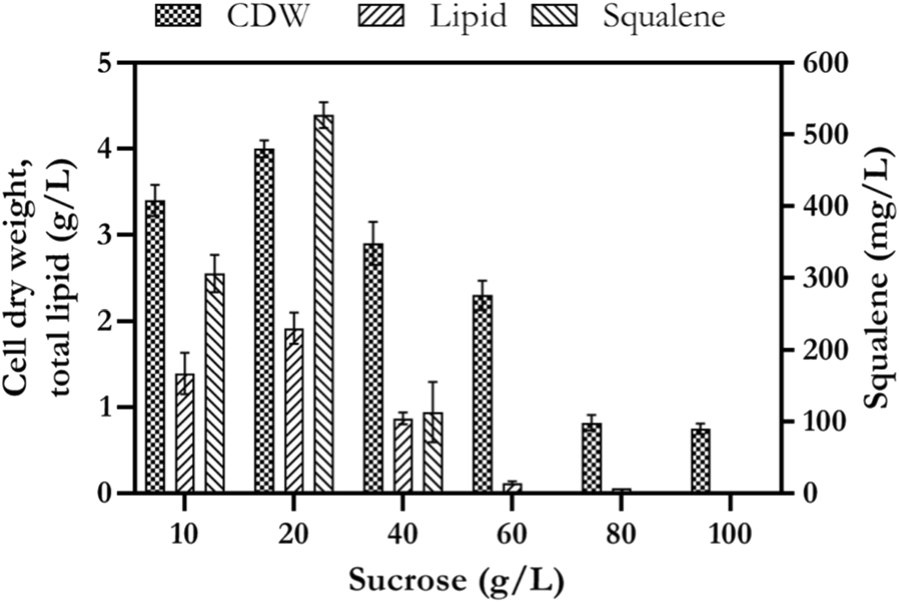
The effect of different concentrations of sucrose on cell growth, lipid and squalene production by *Rhodosporidium* sp. DR37 at 30 °C and 150 rpm

As mentioned before, glucose was a good substrate for production of high amount of biomass, not for squalene accumulation. Therefore, sucrose was selected as proper carbon source for production of higher amount of squalene. So, the effect of various concentrations of sucrose as selected carbon source was investigated on cell growth, lipid and squalene production. As shown in Fig. 7, increase in concentration of sucrose from 10 to 100 g/L significantly dropped production of biomass, lipid and squalene. A sharp decrease in the lipid and squalene production was observed in medium with 60 g/L sucrose. These results showed that growth and lipid production of *Rhodosporidium* sp. DR37 were decreasing while the concentration of sucrose was increasing more than 40 g/L in the culture medium. Jiru et al. showed that optimum glucose concentration for maximum biomass and lipid production by *Rhodosporidium kratochvilovae* SY89 was 50 g/L [63]. Patel et al. showed that the content of polyunsaturated fatty acids such as linoleic acid was significantly higher in *Rhodosporidium kratochvilovae* HIMPA1 while cultivated on medium containing sucrose than medium containing glucose [64]. It could be concluded that sucrose was a favorable carbon source for production of fatty acids and compounds which have more than one double bond in their structure.

### The effect of temperature on cell growth, lipid and squalene production

**Fig. 8:**
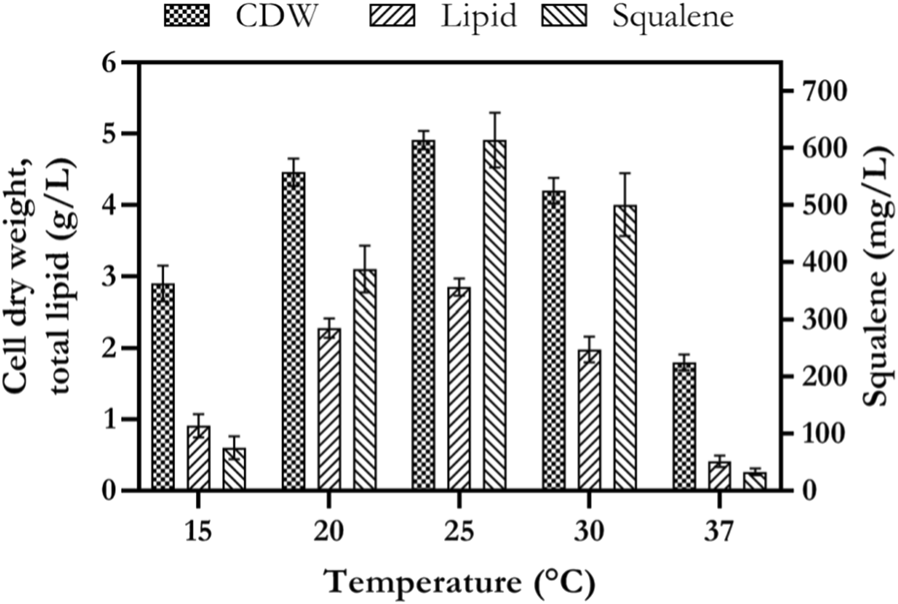
The effect of different temperatures on cell growth, lipid and squalene production by *Rhodosporidium* sp. DR37 at 150 rpm

After determination of proper carbon and nitrogen sources and seawater concentration, temperature and pH were used to test their effects on cell growth, lipid and squalene production by *Rhodosporidium* sp. DR37. The effect of the temperature was tested from 15 to 37 °C. As shown in Fig. 8, an increase in cell growth, lipid and squalene production was observed when the temperature was increased from 15 to 25 °C in culture medium. At 25 °C, biomass, lipid and squalene were 4.9, 2.85 g/L and 614 mg/L, respectively. *Rhodosporidium* sp. DR37 showed its maximum growth and squalene production at 25 °C (*p* < 0.05). Biomass and squalene were decreased to 4.2 g/L and 501 mg/L, when temperature was increased to 30 °C. The minimum level of squalene (33 mg/L) was obtained at 37 °C. Raising temperature to 37 °C showed a statistically significant negative effect on cell growth and squalene production (*p* < 0.05). So, culture medium that was incubated at colder temperature showed best condition for growth and squalene production by *Rhodosporidium* sp. DR37. Actually, a shift to a colder temperature probably can regulate biosynthesis pathways towards squalene and sterol production and accumulation [65]. Also, Wang et al. reported that an increase in temperature from 30 to 35 °C significantly decreased biomass and lipid production by *Rhodosporidium* TJUWZ4 [26]. They reported that temperature range of 20–25 °C is favorable for lipid production by *Rhodosporidium* TJUWZ4. Temperature can regulate sterol biosynthesis pathway in yeasts and subsequently the sterol metabolite profile can change during yeast growth at low (15–25 °C) or high temperatures (30–37 °C) [65].

### The effect of initial medium pH on cell growth, lipid and squalene production

**Fig. 9:**
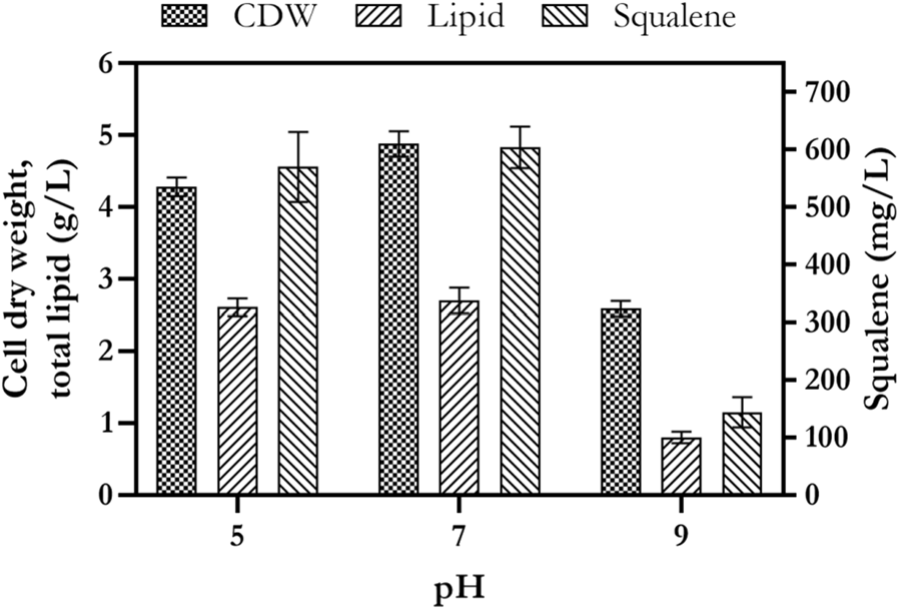
The effect of various pH on cell growth, lipid and squalene production by *Rhodosporidium* sp. DR37 at 25 °C and 150 rpm

As shown in Fig. 9, it was found that the initial medium pH 5 and 7 had similar effects on cell growth, lipid and squalene production. The amounts of biomass, lipid and squalene were 4.28, 2.61 g/L and 570 mg/L at pH 5, and 4.88, 2.7 g/L and 604 mg/L at pH 7, respectively. But at initial medium pH 9, a significant decrease (*p* ˂ 0.05) in cell growth, lipid and squalene was observed to 2.59, 0.8 g/L and 144 mg/L, respectively. *Rhodosporidium* sp. DR37 showed good adaptation to initial medium pH 5 similar to pH 7. Increasing of pH to 9 had a negative effect on cell growth and squalene production in comparison to pH 5 and 7. These results are in accordance with Wang et al. [26]. Their results showed that two strains of *Rhodosporidium* TJUWZ4 and *Rhodosporidium* TJUWZA11 were able to accumulate lipid in media with initial pH range of 3–7. They concluded that this acid tolerance property makes both strains good candidates for high lipid production in media with initial low pH. Therefore, the presence of initial low pH in culture medium is a good factor for prevention of microbial contaminations in fermentation broth.

**Fig. 10:**
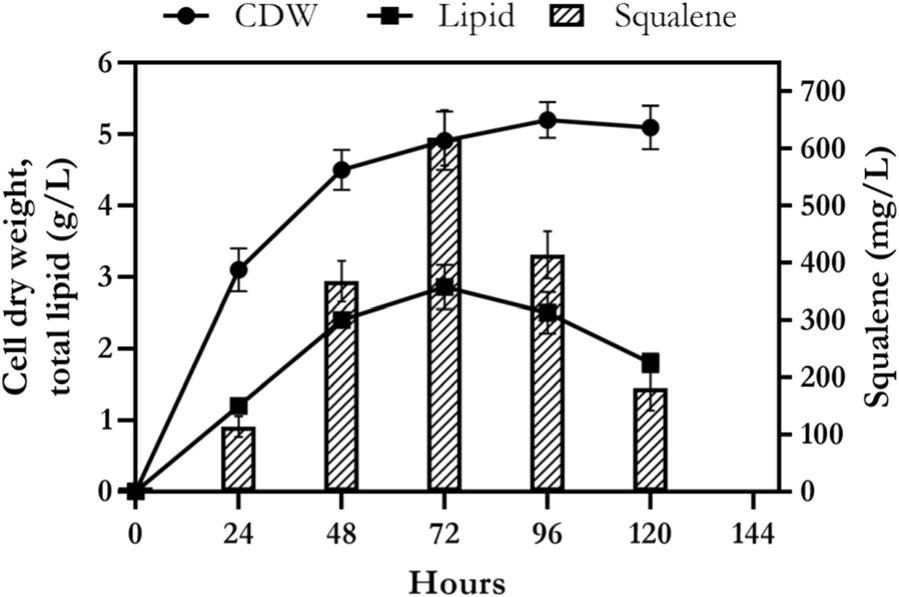
Biomass, lipid and squalene production profile of *Rhodosporidium* sp. DR37 cultured in optimized medium at 25 °C for 120 h at 150 rpm. Lipid and squalene yields were measured as g/L and mg/L of cell culture, respectively. The values are the mean ± standard deviation of three replicates

Finally, the optimum culture condition was included 20 g/L sucrose, 5 g/L peptone, 20% v/v seawater and pH 7 at 25 °C. Cell growth, lipid and squalene production were monitored in optimized shake-flask culture condition during 120 h. As shown in Fig. 10, the amount of squalene was increasing during 72 h of cultivation. The maximum amount of lipid (2.86 g/L) and squalene (619 mg/L) was achieved at 72 h of incubation. Both lipid and squalene production were decreased after 72 h and reached to 1.8 g/L and 181 mg/L at 120 h, respectively.

**Fig. 11:**
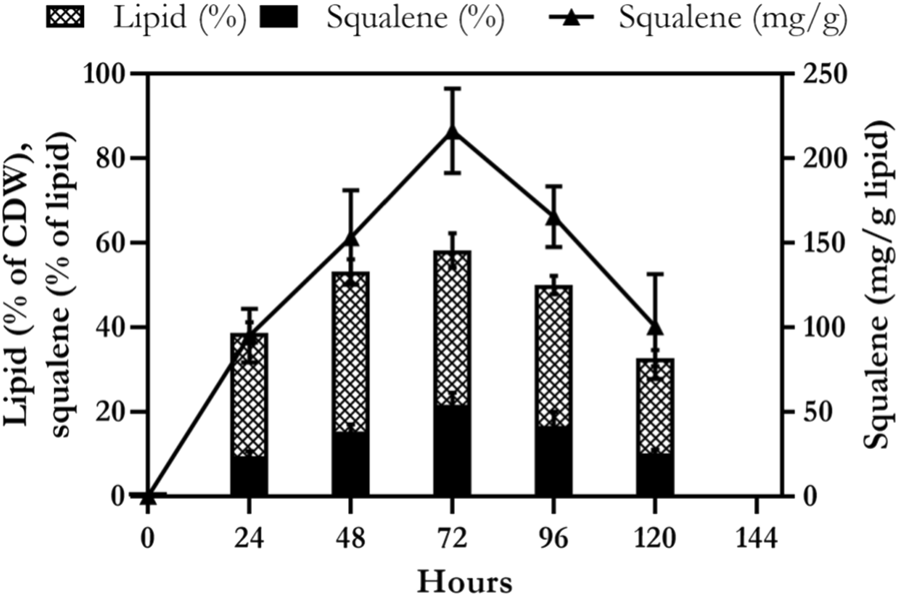
Profile of lipid and squalene content of *Rhodosporidium* sp. DR37 cultured in optimized medium at 25 °C for 120 h at 150 rpm. Lipid and squalene content were measured as % of CDW and total lipid, respectively. Also squalene content mg/g CDW was determined. The values are the mean ± standard deviation of three replicates

As shown in Fig. 11, lipid content (% of CDW) and squalene content (% of total lipid) in *Rhodosporidium* sp. DR37 cultivated in optimized medium were investigated by time course analysis. The lipid content was 38.7% and 53.3% at 24 and 48 h, respectively, and reached to its maximum level (58.2%) at 72 h. A similar pattern was observed for squalene content. The maximum content of squalene (21.6%) was achieved at 72 h and then decreased to 10.1% at 120 h.

### Comparison of kinetic parameters in production and stationary phases of *Rhodosporidium* sp. DR37

As shown in Fig. 10, profile of cell growth, lipid and squalene production in *Rhodosporidium* sp. DR37 can be divided into two phases. The first stage represents the production phase (0–72 h) and the second stage refers to the stationary or consumption phase (72–120 h). Table 2 shows kinetic parameters of production phase (0–72 h) and stationary phase (72–120 h) of *Rhodosporidium* sp. DR37 in optimized medium. Kinetic parameters including *μ*_*max*_ (h^−1^), *q*_*s*_ (h^−1^), *q*_*p*_ (h^−1^), *q*_*sq*_ (h^−1^), *Y*_*x/s*_ (%), *Y*_*p/s*_ (%) and *Y*_*sq/s*_ (%) were calculated and compared during these two phases. At the production phase, the value of *μ*_*max*_, *q*_*s*_, *q*_*p*_ and *q*_*sq*_ were obtained 0.092, 0.226, 0.036 and 0.010, respectively. Also, *Y*_*x/s*_, *Y*_*p/s*_ and *Y*_*sq/s*_ were calculated as 28.1, 16.3 and 3.5, respectively. A sharp increase and maximum level of biomass, lipid and squalene was observed at the end of this phase. As shown in Table 2, the values of *Y*_*x/s*_, *Y*_*p/s*_ and *Y*_*sq/s*_ in the stationary phase were decreased to 11.9, -66.3 and -27.4, respectively. The significant decrease and difference (*p* < 0.05) in *Y*_*x/s*_, *Y*_*p/s*_ and *Y*_*sq/s*_ after 72 h demonstrate that stationary phase was not only favorable for lipid and squalene production, but also accelerated their consumption. On the other hand, biomass, lipid and squalene were produced in high amount during exponential growth phase. Therefore, it can be mentioned that production of lipid and squalene is growth-dependent in *Rhodosporidium* sp. DR37. The significant decline in the values of *Y*_*x/s*_, *Y*_*p/s*_ and *Y*_*sq/s*_ in the stationary phase, demonstrated the high lipid and squalene consumption rate during this phase. As most of the carbon source was consumed after 72 h (data not shown), accumulation of lipid and squalene was stopped and the content of intracellular lipids decreased rapidly between 72 and 120 h. It could be mentioned that lipid content can be used as a source of energy storage during starvation. Taking into account the cell starvation in stationary phase, acceleration of lipid and squalene consumption can provide more energy for cellular activities and maintenance. Guerreiro et al. [66] obtained *Y*_*p/s*_ of 0.03 g/g when *Rhodosporidium* toruloides CECT 1499 was cultivated in shake flask with lipid production medium containing 13 g/L glucose and 0.75 g/L YE at 150 rpm and 30 °C for 146 h. Another research work on *Rhodosporidium* toruloides NCYC 921 has reported *Y*_*p/s*_ of 0.08 and 0.13 g/g in carbon or nitrogen-limiting batch medium in bioreactor, respectively [67]. These obtained yields were lower than obtained *Y*_*p/s*_ by *Rhodosporidium* sp. DR37, indicating optimized medium supports the growth of *Rhodosporidium* sp. DR37 and subsequent accumulation of lipid and squalene during production phase.

**Table 2:** Comparison of the kinetic parameters of *Rhodosporidium* sp. DR37 at production phase (0–72 h) and stationary phase (72–120 h) in optimized medium

Kinetic parameters	Optimized medium	
	Production phase (0–72 h)	Stationary phase (72–120 h)
*μ*_*max*_ (h^−1^)	0.092	–
*q*_*s*_ (h^−1^)	0.226	–
*q*_*p*_ (h^−1^)	0.036	–
*q*_*sq*_ (h^−1^)	0.010	–
*Y*_*x/s*_ (%)	28.1^a^	11.9^b^
*Y*_*p/s*_ (%)	16.3^a^	− 66.3^b^
*Y*_*sq/s*_ (%)	3.5^a^	− 27.4^b^

Different letters (a, b) in the same row show significant difference between the parameters (*p* ˂ 0.05). *μ*_*max*_ maximum specific cell growth rate, *q*_*p*_ specific lipid accumulation rate, *q*_*sq*_ specific squalene accumulation rate, *q*_*s*_ specific sucrose consumption rate, *Y*_*x/s*_ growth yield, *Y*_*p/s*_ lipid yield, *Y*_*sq/s*_ squalene yield

It has been reported that two strains of *Pseudozyma* and three strains of *Aurantiochytrium* are able to produce significant amount of squalene (Table 3). The final yield of 1290 mg/L with content of 33.07% was achieved for production of squalene by *Aurantiochytrium* 18 W-3a [38]. Another research work on this strain has reported yield and maximum content of squalene as 900 mg/L and 68.99%, respectively [17]. It has been reported that the highest content of squalene was achieved by *Aurantiochytrium* Yonez5-1 as 74.05% with yield of 1073.66 mg/L [13]. In this study, *Rhodosporidium* sp. DR37 showed squalene yield of 619 mg/L and content of 21.6%. Also, two strains of *Pseudozyma* were reported to be able to produce 340–2445 mg/L of squalene.

**Table 3:** Comparison of the squalene yield and content by *Rhodosporidium* sp. DR37 mentioned in this study

Strain	Squalene yield (mg/L)	Shake-flask studies	Cultivation mode	References
		Squalene content (% of lipid)		
*Aurantiochytrium* 18 W-3a	1290	33.07%	Shake flask	[38]
*Aurantiochytrium* Yonez5-1	1073.66	74.05%	Shake flask	[13]
*Aurantiochytrium* 18 W-3a	900	68.99%	Shake flask	[17]
*Pseudozyma* sp. JCC 207	340.52	NR	Shake flask	[23]
*Pseudozyma* sp. SD301	2445	NR	Fed-batch fermentation	[16]
*Rhodosporidium* sp. DR37	619	21.6%	Shake flask	This study

*NR* not reported

The yield of the produced squalene by *Rhodosporidium* sp. DR37 was 1.8 times more than produced squalene by *Pseudozyma* sp. JCC 207 [16] and 3.9 times less than produced squalene by *Pseudozyma* sp. SD301. It must be considered that 2445 mg/L produced squalene by *Pseudozyma* sp. SD301 was achieved in fed-batch fermentation in a 5-L bioreactor [23], while 619 mg/L produced squalene by *Rhodosporidium* sp. DR37 was obtained in a shake-flask cultivation medium.

## Conclusion

Lipid and carotenoids production has been widely studied in *Rhodosporidium* species. This oleaginous yeast strain is currently being used for production of lipids in large-scale bioreactors. However, in this study, we demonstrated that *Rhodosporidium* sp. DR37 is also a favorable and promising candidate for production of squalene. By cultivation of *Rhodosporidium* sp. DR37 in optimized medium, highest squalene accumulation (619 mg/L) and content (21.6% of total lipid) were achieved. Moreover, *Rhodosporidium* sp. DR37 was able to produce significant amount of squalene in a wide range of seawater concentration (0–50% v/v), temperature (20–30 °C) and pH (5–7). Although, the highest amount of squalene was achieved in optimized medium with 20% v/v seawater, 25 °C and pH 7. Comparison of *Y*_*x/s*_, *Y*_*p/s*_ and *Y*_*sq/s*_ during growth of *Rhodosporidium* sp. DR37 in optimized medium revealed that lipid and squalene were accumulated in production phase and then consumed in stationary phase. Interestingly, cultivation and growth of *Rhodosporidium* sp. DR37 on medium containing sucrose as sole carbon source indicated that squalene can be co-produced with other high-value metabolites such as carotenoids from consumption of cost-effective and cheap feedstocks. Therefore, these results prove further investigations on squalene production in fed-batch fermentation in bioreactor which is the subject of our forthcoming research work.

## Data Availability

All data generated or analyzed during this study are included in this published article.
